# The Medical Student's Complaint

**Published:** 1887-04-30

**Authors:** 


### THE MEDICAL STUDENT'S COMPLAINT.

I do not like the taste of beer,
Tobacco makes me sick,
The headache whisky leaves I fear,
I hate a heavy stick;
And yet I always smoke and drink,
And take where er I go
My heavy stick, because I think
That students all do so.

Billiards I do not understand,
At cards I am a fool,
But yet I take a cue in hand
At pyramids or pool;
At nap I've lost my ready cash,
A good deal more I owe.—
Perhaps such conduct's wild and rash,
But students all do so.

I do not like the music-hall;
The songs that make folks laugh
I cannot understand, nor all
The stuff that they call chaff.
The glare, the heat, the gas I hate,
Yet there I often go,
Though quite done up with hours so late,
For students all do so.

I don't think it a clever thing
In class to make a noise:
To shout and whistle, stamp and sing,
Is only fit for boys;
Yet now, by dint of practice great,
Just like a cock I crow;
It makes the lecturer irate,
So students all do so.

At home I never used to swear,—
They would have thought it wrong;
But here of course I need not care,
Although my speech is strong.
I speak the strongest oaths quite pat,—
They're out before I know;
I don't half like it,—what of that,
When students all do so?

Religion never gave me doubt,
I thought the parson knew
What all these questions were about,
And told us what was true:
But here the Bible's dubbed untrue,
"Agnosticism's" the go;
So I must play the sceptic too,
For students all do so.

I'm ruining my health, I fear,
With staying out at night;
My brain is never very clear,
My eyes are never bright;
I'm not so strong as in time past,
My stomach's but so-so;
I'd rather not live quite so fast,
But students all do so.

Since I to college came I've tried
To do as others do;
And now the truth I cannot hide,
Exams. I'll ne'er get through;
For if I ever try to read
I'm voted "precious slow";
I'd rather study hard, *indeed*,
But—students don't do so.

